# Cyclic undecapeptide Cyclosporin A mediated inhibition of amyloid synthesis: Implications in alleviation of amyloid induced neurotoxicity

**DOI:** 10.1038/s41598-018-35645-4

**Published:** 2018-11-23

**Authors:** Shadab Kazmi, Anzar Abdul Mujeeb, Mohammad Owais

**Affiliations:** Molecular Immunology Laboratory, Interdisciplinary Biotechnology Unit, Aligarh Muslim University, Aligarh, 202002 India

## Abstract

Amyloids are highly organized fibril aggregates arise from inappropriately folded form of the protein or polypeptide precursors under both physiological as well as simulated ambience. Amyloid synthesis is a multistep process that involves formation of several metastable intermediates. Among various intermediate species, the as-formed soluble oligomers are extremely toxic to the neuronal cells. In the present study, we evaluated cyclosporine A (CsA), an undecapeptide, for its potential to prevent aggregation of model protein ovalbumin (OVA). In an attempt to elucidate involved operative mechanism, the preliminary studies delineate that CsA affects both primary nucleation as well as other secondary pathways involved in OVA fibrillation process. By its specific interaction with amyloid intermediates, the cyclic peptide CsA seems to regulate the lag phase of the fibrillation process in concentration dependent manner. The present study further suggests that exposure to CsA during lag phase ensues in reversal of OVA fibrillation process. On the contrary, mature OVA fibril remained impervious to the CsA treatment. The cyclic undecapeptide CsA was also found to successfully alleviate amyloid induced toxicity in neuroblastoma cells.

## Introduction

Aggregated forms of protein amyloids have a great deal of correlation with a growing list of diseases, including diabetes, Alzheimer’s, Parkinson’s, Huntington’s and Prion disease etc^1^. During fibrillogenesis, misfolded proteins transform into a long, unbranched, β–sheet rich fibrillar structure^2^. Protein aggregation generally ensues in generation of amyloid species in both *in vitro* as well as *in vivo* conditions^3^. In general, native proteins keep aggregation-prone residues buried in the hydrophobic core to avoid aggregation^4^. The self-assemblage of β-strand forming short, aggregation-prone amino acid residues, is supposed to result in the formation of protein aggregates^5^ that may often acquire toxic properties^6^.

Amyloid-beta (Aβ) peptides (comprised of 39–43 amino acid residues) are main components of amyloid plaques^7^. They have been widely implicated in both familial as well as sporadic Alzheimer’s disease. The amyloid cascade hypothesis presumes that amyloid aggregates, self-assembled from misfolded Aβ peptides, affect the structure and function of neuronal cells and stimulate apoptosis^8,9^. This eventually results into synaptic dysfunction and neurodegeneration^10^.

By stabilizing native state, small molecules/peptides can hinder the fibrillation process and also reverse the misfolding of the protein thus can serve as promising clinical agent against many associated debilitating diseases^11,12^. The observation is substantiated from the fact that there is a remarkable increase in the number of research articles, during recent past, reporting short peptides mediated inhibition of amyloid aggregation^12–14^. Recent advent in molecular biology and peptide synthesis technology has made it possible to fabricate specific peptides that have potential to inhibit aggregation. While it remains to be seen whether short peptides can be exploited as therapeutic agents to prevent the amyloid related diseases, nonetheless, such inhibitors can help us to comprehend intricacies associated with protein aggregation process^14^. The short peptide based inhibitors can also be helpful in deciphering the characteristics of prospective therapeutic agents with amyloid inhibition properties^15^. It is speculated that they inhibit protein misfolding by stabilization of the native structure.

Besides small sized peptides, cyclic peptides have also been found to inhibit or modify the self-assembly of aggregation-prone sequences^16^. In the present study, we have elucidated the effect of a medically relevant cyclic peptide, cyclosporine A (CsA), on inhibition of amyloidogenesis. CsA is widely used as an immunosuppressive drug^17^. It is a cyclic undecapeptide (molecular weight 1202.6) obtained by fermentation of two fungi, Trichoderma polysporum and Cylindrocarpon lucidum^17^. It crosses the blood brain barrier (BBB) and has potential to enhance the proliferation and survival of neural precursor cells in both *in vitro* and *in vivo* conditions^18^. CsA imparts neuroprotection against neural trauma in animal models by alleviating mitochondrial dysfunction and attenuating axonal disruption^19^. It is also used as immunosuppressive agents during organ transplantation^17^.Upon administration, 90% of CsA remains bound to lipoproteins^20^. It can also bind to albumin, globulins and various other protein of the blood^21^. While in the systemic circulation, CsA can also extensively associate with erythrocytes^21^. The binding with erythrocytes can affect its bioavailability. The pharmacokinetic profile of CsA is capricious and can vary from population to population^22^. CsA may be administered as a continuous infusion, once per day, or twice a day with extended infusion times (2–13 h per day). Target dosing range varies between 1 and 20 mg/kg per day and is generally implemented to attain blood plasma levels, that ranges between 100 and 1000 ng/ml^23^. A dose of 100–500 ng/ml of CsA had been employed to study effect of CsA treatment on neurosphere cells^24^.

Besides its specific effect on host immune system, CsA has been reported to inhibit Cyclophilins (CyP)^25^ as well. Cyclophilins are ubiquitously distributed proteins belonging to the immunophilin family^25^. CyP has peptidyl prolyl cis-trans isomerase (PPIase) activity, which regulates protein folding and trafficking^26^. Although CyP was initially believed to function primarily as an intracellular protein, recent studies have revealed that it can be secreted by cells in response to inflammatory stimuli as well^27^. Current research in both animal models and humans has provided compelling evidences highlighting the critical function of CyP in Alzheimer’s disease^25^. CsA binds both extracellular and intracellular CyP and inhibits its PPIase activity^26^. In particular, it inhibits the protein phosphatase calcineurin and blocks the translocation of NF-AT nuclear factor from the cytosol to the nucleus, thus prevent the transcription of the genes encoding pro-inflammatory cytokines. Yurchenko *et al*. reported that CD147 interacts with extracellular CyPA in a CsA-sensitive fashion^28^.

Traumatic Brain Injury (TBI) has devastating acute manifestations and may cause alterations in protein folding, protein binding, clearance of many therapeutic agents and their metabolism^29–31^. TBI increases the propensity of developing Alzheimer’s disease (AD) in later part of the life^32^. Although the fundamental mechanism is not known, it has been reported that TBI promotes accumulation of Aβ in axons of the injured brain^33^ and also causes deposition of insoluble Aβ plaques^34^. In TBI patients, CsA is cleared more rapidly and has a larger distribution volume (Vd) as compared to non-TBI population^35^. The physiochemical properties of CsA limit its penetration into the central nervous system (CNS) under normal physiological conditions^34^, however, post TBI disruption, a biphasic opening is created which influences a window of opportunity for CsA to gain access to the injured brain^36^.

Considering the fact that attainment of minimal effective (systemic/or CNS) concentration of a theraputeic agent is a key factor in optimizing treatment strategy of TBI, we explored potential of varying amount of CsA to inhibit protein aggregation process. Next, we tried to decipher role of CsA in modulating specific underlying molecular processes involved in CsA mediated inhibition of amyloid formation. The effect of CsA on fibrillation of model protein OVA was assessed employing techniques such as Thioflavin T (ThT) and Congo Red (CR) binding as well as CD spectral measurements mainly. The formation of soluble fibrils was confirmed by transmission electron microscopy (TEM). We also studied the potential of CsA to alleviate OVA fibril induced toxicity in neuroblastoma cells.

## Results

### CsA regulates aggregation process of soluble OVA

Relative Fluorescence intensity (FI) has been widely exploited to study protein aggregation. In fact changes in extrinsic fluorophore associated florescence intensity have been considered as an efficient tool to elucidate the possible mechanism operative in protein aggregate synthesis.

**Figure 1:**
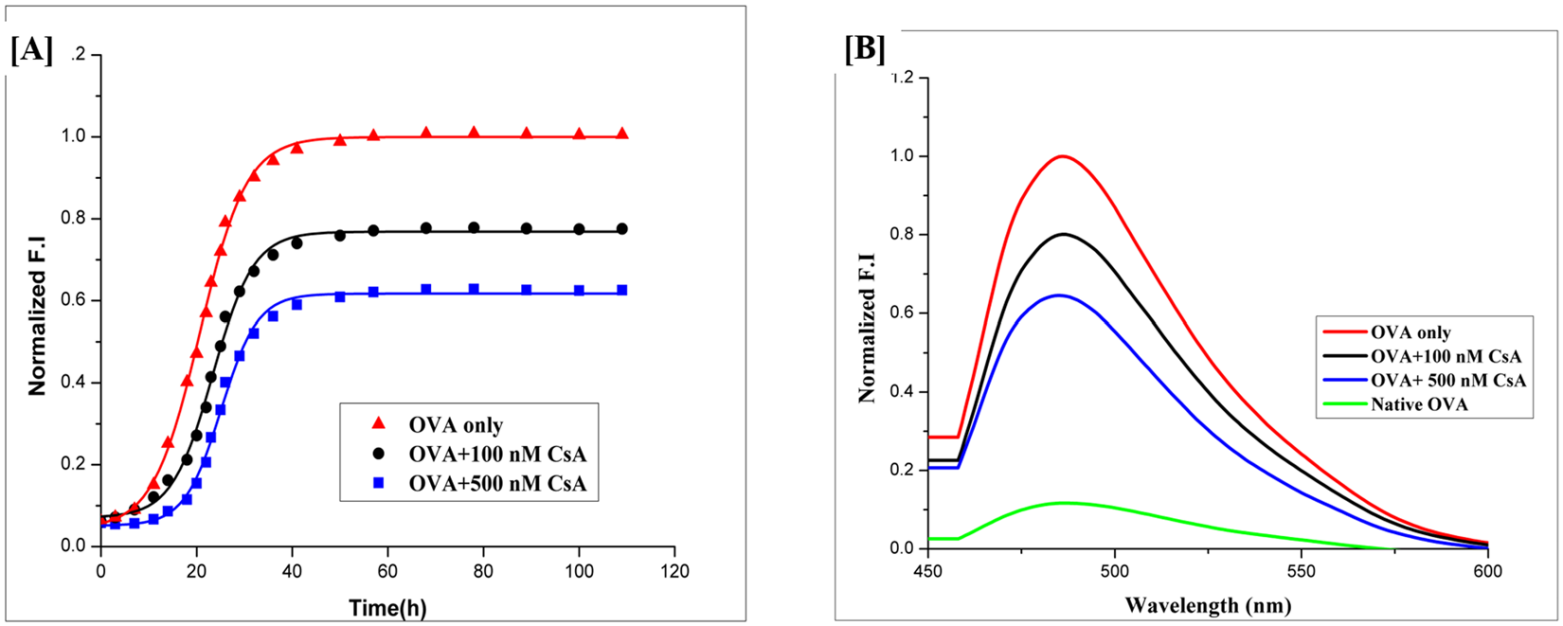
Effect of cyclic peptide CsA on kinetics of OVA amyloidogenesis. (**A**) The effect of CsA on synthesis of OVA amyloid was monitored by determining ThT fluorescence associated with as-formed OVA amyloid. The OVA amyloid synthesis followed a sigmoidal kinetics that involves around 5 h lag time before attainment of exponential phase. The cyclic peptide CsA inhibits OVA-amyloid synthesis in concentration dependent manner. (**B**) Absorption fluorescence spectra of ThT bound mature OVA amyloid species generated at 120 h time point. The effect of presence of CsA on generation of OVA amyloid was monitored by plotting fluorescence absorption spectrum of mature OVA amyloid formed at 120 h time point. The CsA inhibited OVA amyloid synthesis in dose dependent manner. The native ova failed to bind with ThT dye.

In the present study, ThT dye associated relative fluorescence intensity (RF intensity) has been used as a parameter to investigate the effect of CsA on OVA fibril synthesis^37^. The ThT dye specifically interacts with crossed-β sheet structure of as-synthesized fibril that eventually result in a significant rise in relative fluorescence intensity (RF value) of the binding complex (Fig. 1A). The arbitrary RF value of bound ThT dye decreased from 222 to 96 in presence of 100 nM CsA. It further lowered down to arbitrary RF value 50 in presence of 500 nM CsA. The plain OVA when allowed to interact with ThT in presence of CsA did not induce ThT fluorescence (Fig. 1B). Interestingly, addition of CsA to preformed OVA amyloid fibrils did not cause any change (decrease or increase) in ThT fluorescence intensity. The observation suggests that CsA cannot revert already formed fibril, rather, it can inhibit fibril formation only.

### Effect of CsA on induction of OVA amyloid synthesis

**Figure 2:**
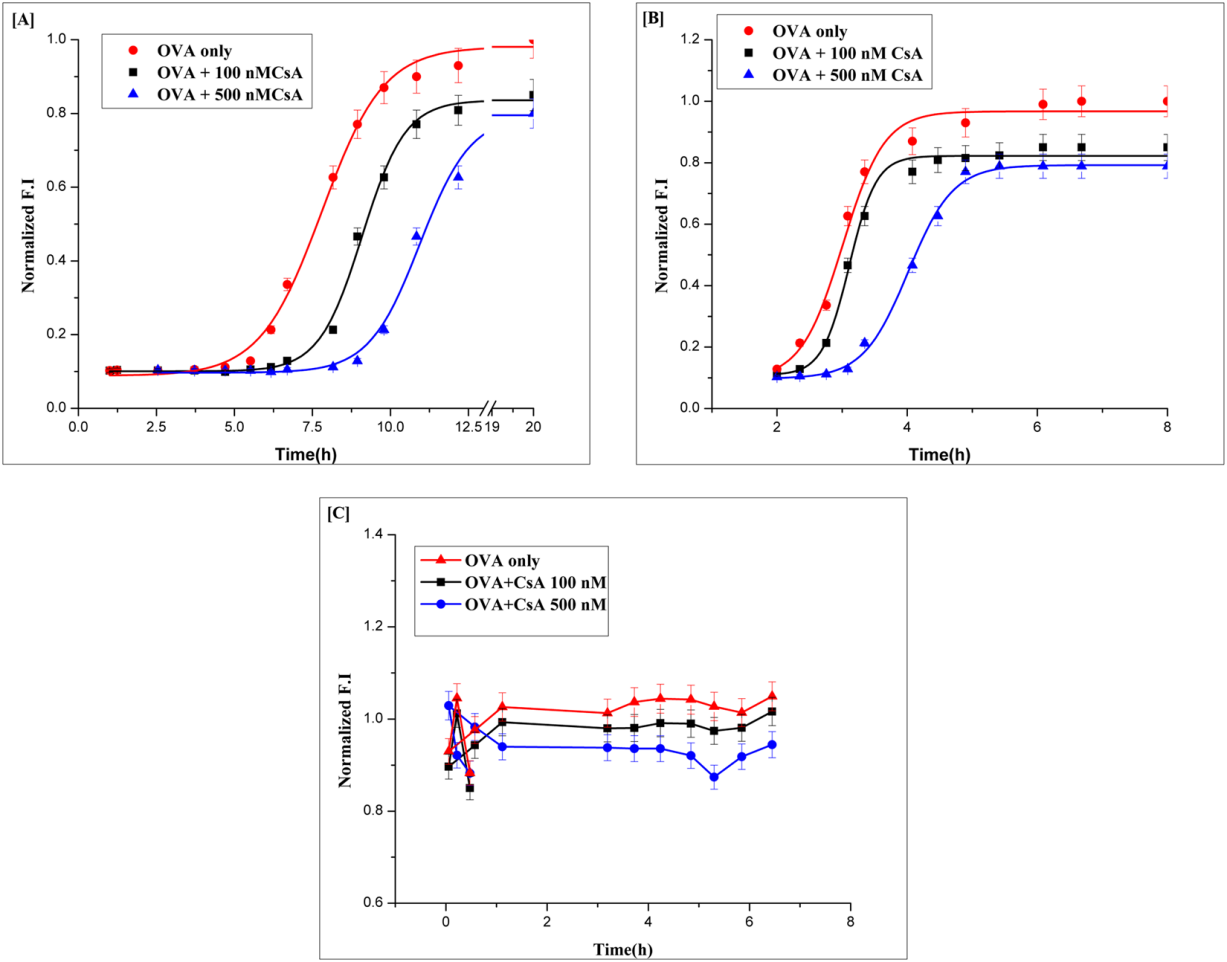
Effect of Cyclic peptide CsA on chemical kinetics of OVA aggregation. The effect of CsA on chemical kinetics of OVA fibrillation was monitored by determining ThT fluorescence associated with as-formed OVA fibril at various time points. (**A**) Increasing concentration of CsA was added to the reaction mixture at 1 h post beginning of the incubation (cf. lag phase). (**B**) CsA was added to the reaction mixture in concentration dependent manner at 5 h post beginning of the incubation (cf. exponential growth phase). (**C**) Increasing concentration of CsA was added to the reaction mixture at 18 h post beginning of incubation (*cf*. saturation phase). Red line in each of the panels corresponds to the aggregation profile of OVA that was not exposed to CsA and served as control. The volume of the control setup was maintained by addition of plain buffer only.

The basic understanding of chemical kinetics of amyloid synthesis on one hand, and an insight of involved nucleation process on the other, can generally help in elucidation of underlying operative mechanism responsible in CsA mediated blockade of OVA amyloidogenesis. The aggregation kinetics of OVA fibrillation exhibited a typical sigmoidal curve consisting of a nucleation process involving lag phase (upto 300 minutes), followed by a rapid growth phase associated with the elongation and propagation of fibril synthesis eventually a final stationary phase^38^. In general, protein aggregation process involves three different successive phases viz. lag phase, exponential growth phase, and stationary phase. Lag-phase is not associated to primary nucleation only, but also to elongation and growth stages as well. Thus, at least two viz. primary nucleation; and elongation; and in many systems at least four viz. primary nucleation, elongation, secondary nucleation and fragmentation involving microscopic processes may occur during the lag phase. It seems all above specified processes may occur during each of the three phases of the macroscopic aggregation process as well, albeit at different rates that is generally regulated by both rate constant as well as the concentration of reacting species at specific time point^39^. The interaction of CsA with early intermediates was substantiated by ThT binding assay. Further, incubation of OVA with ThT beyond 300 minutes resulted in enhanced relative fluorescence intensity, indicative of amyloid fibril formation. In order to assess the potential of CsA to inhibit protein aggregation, it was added to the unseeded reaction mixture at various time points. An equivalent amount of buffer was added in the control experiment, to nullify the effect of dilution. The addition of CsA in mid of the lag phase (1 hour post start of the unseeded reaction), was found to increase lag phase by several minutes, as opposed to the control reaction with the lag phase of around 300 minutes (5 h) (Fig. 2A). It seems CsA delayed the time lapse that can be exploited by various as-formed species to acquire potential to enter the growth phase. The observation suggest that CsA played a vital role in inhibiting the early intermediates, which remain no more available to form the elongation competent species. Addition of CsA at the end of the lag phase (at 5 hours time point of an unseeded reaction), led to slight increase in lag phase. The aggregate growth proceeded with a very gentle slope (Fig. 2B). The extent of inhibition was greater in the situation when CsA was added during the lag phase. Addition of CsA at later time points (for instance during the end of growth phase) did not affect fibril synthesis (Fig. 2C). CsA was not able to disaggregate the already formed amyloid. The results are also suggestive of the fact that CsA is most effective in preventing the aggregation with extended and prolonged lag phase. The observation suggests that, CsA delays the aggregation reaction by inhibiting primary nucleation. The present set of experiments does not provide information regarding relative contributions of secondary nucleation as well as other secondary pathways that contribute in overall aggregation reaction.

**Figure 3:**
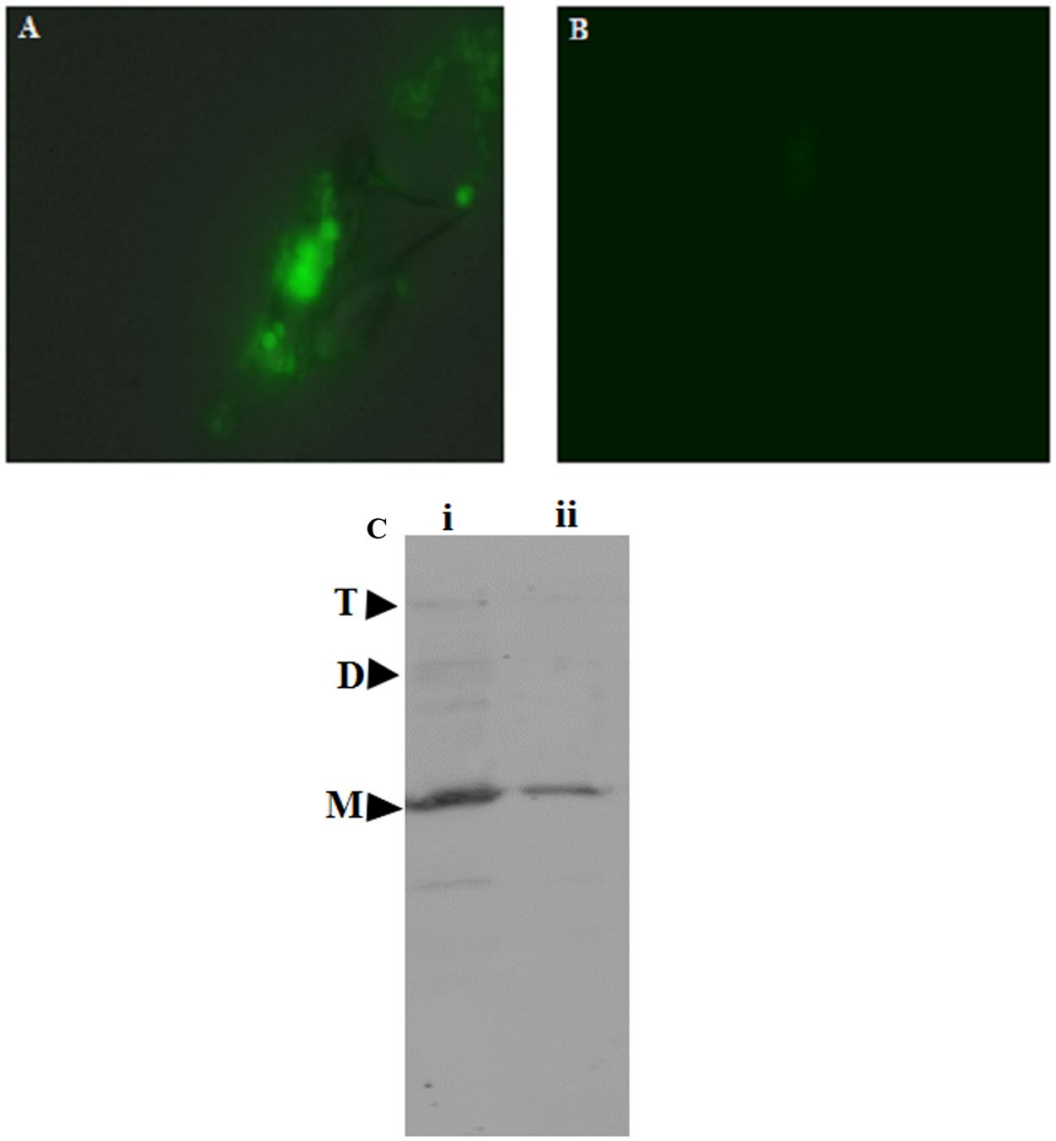
Cyclic peptide CsA mediated inhibition of OVA amyloid synthesis as revealed by fluoresense microscopy. Green fluorescence associated with OVA amyloid bound ThT was visualized employing epifluoresense microscopy. The epi-fluoprescence micrographs of the as-synthesized OVA amyloid formed in absenccse (**A**) or presense (**B**) of 500 nM CsA. The panel (C) corresponds to Western blot profile of amyloid intermediates formed during fibril formation. Western blot analysis of OVA-amyloid probed with OVA specific 3G2E1D9 monoclonal antibodies reveals presence of monomeric, dimeric as well as oligomeric OVA (lane i) formed at the beginning of exponential phase of OVA aggregation process (without CsA). Lane ii shows inhibitory effect CsA (500 nM) on the formation of monomeric and dimeric intermediates of the OVA.

CsA mediated inhibition of amyloid formation was further confirmed by employing fluorescence microscopy, which showed minimal aggregate formation when OVA was allowed to stir in the presence of CsA (Fig. 3A,B). Further, CsA failed to revert or lower down ThT emission upon its incubation with the preformed OVA fibrils (data not shown). Taken together, the data show that CsA, at sub-stoichiometric ratios, inhibit formation of OVA fibrillation in a concentration-dependent manner.

### CsA inhibits synthesis of OVA oligomers

The effect of CsA mediated inhibition of OVA amyloid was also followed by specific interaction of various as-formed species generated during amyloid formation cascade. The time kinetics of fibril bound ThT fluorescence followed a typical sigmoidal curve. Immunoblots co-incubated with conformational sensitive anti-OVA 3G2E1D9 monoclonal antibody showed presence of OVA oligomers at 5 h time point in absence of CsA (corresponding to lane i) (Fig. 3C). No oligomeric band was observed in lane ii that corresponds to reaction mixture incubated for 5 h in presence of CsA. The result suggested that CsA inhibit OVA fibrillation when added during lag phase.

### Effect of CsA on secondary pathways operative in OVA aggregation process

**Figure 4:**
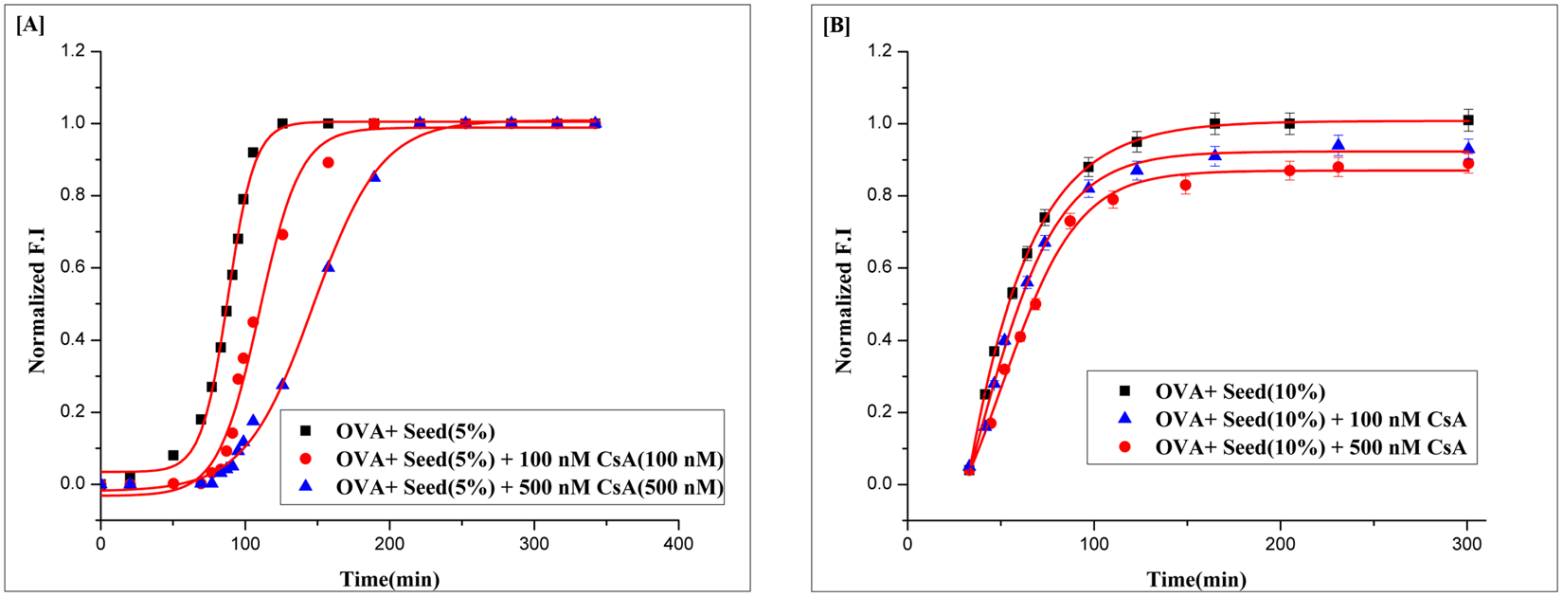
(**A**) Role of CsA on inhibition of secondary pathway of OVA fibril synthesis. Effect of 5% pre-formed fibril seeds on aggregation of OVA(1 mg/ml) in the presence of cyclic peptide CsA. (**B**) Effect of 10% fibril seeds (pre-formed) on Aggregation of OVA(1 mg/ml) in the presence of increasing concentrations of CsA.

To further strengthen the proposed hypothesis involved in CsA mediated inhibition of fibril synthesis, we studied the aggregation kinetics of OVA in presence of fibril seeds (preformed digested fibril) introduced to the reaction mixture at the time of incubation. The aggregation kinetics of OVA amyloid synthesis bypassed lag phase in the presence of fibril seeds (Fig. 4A,B). In such circumstances, the contribution of primary nucleation to the reaction kinetics is likely to be inconsequential, as the conversion of soluble OVA into mature fibrils will now be greatly accelerated by secondary nucleation. For example, in the presence of 10% fibril seeds, where preformed fibril seeds mediated fibrillation is the dominant mechanism, no effect of CsA (upto 500 nM) was observed on OVA aggregation kinetics (Fig. 4B). In contrast, corresponding OVA aggregation setup with no preformed fibril (unseeded) was delayed for several minutes in the presence of 100 nM CsA. Furthermore, we also measured the kinetics of OVA fibrillation (1 mg/ml) in the presence of 5% preformed fibril seeds (Fig. 4A). Under these conditions, the primary nucleation was almost bypassed, whereas both surface -catalyzed secondary nucleation and elongation significantly contribute to the overall kinetics (i.e. secondary pathways) (Fig. 4B). On the basis of the above set of experiments, we infer that CsA causes decrease in the rate of surface-catalyzed secondary nucleation. We can also speculate that although the elongation of fibrils remains unaffected essentially, the presence of CsA effectively modulate both primary and secondary nucleation pathways involved in OVA aggregation. The observation that CsA inhibits OVA aggregation by specifically perturbing either of the operative pathways could result from the interaction of CsA with monomers, in principle, or with primary and secondary oligomers, or indeed with both monomers and oligomers simultaneously.

### CsA inhibits amyloid fibrillation of Aβ-42 peptide as well

The Aβ-42 fibrillation was followed for a period of 24 h. The fluorescence of ThT bound Aβ-42 was found to increase in time dependent manner. CsA mediated inhibition of Aβ-42 fibrillation was also examined in presence of increasing amount of the drug. Fig. S1 represents the ThT kinetics of Aβ-42 aggregation. ThT fluorescence intensity of the aged amyloids demonstrated strong ThT emission. The aggregation of Aβ-42 was reduced in the presence of 100 nM CsA (Fig. S1). A relatively large amount of CsA (500 nM) inhibits fibrillation of Aβ-42 to a greater extent (Fig. S1).

### Congo red binding assay further corraborates anti-fibril potential of cyclic peptide CsA

**Figure 5:**
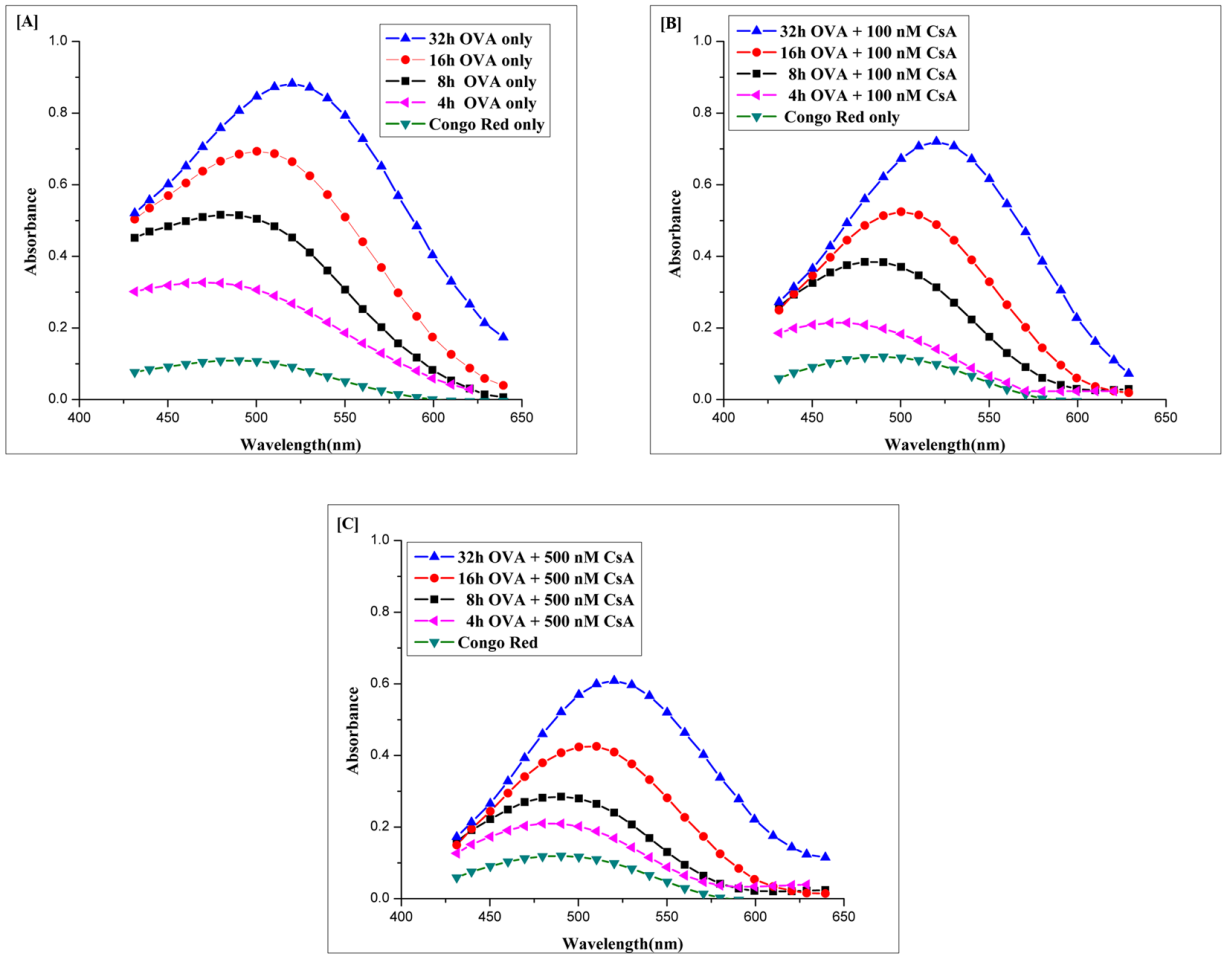
Absorption spectra of Congo Red bound OVA fibril. As-synthesized OVA amyloid samples were incubated with congo red dye. The corresponding absorption spectra was plotted as a function of absorbance for OVA only (without CsA) (**A**), and two different concentration of CsA 100 nM (**B**) and 500 nM **(C**) at 25 °C for various time points. CR solution (20 μM) in PB served as a control (CR-only spectrum). The absorbance of 100 μg of native OVA mixed with 20 μM of CR solution was also measured. The CsA was found to reinstate red shift in as-formed OVA-fibril. Experimental data represent of means of three determinants ± S.D (n = 3). The data is representative of three different experiments with same pattern.

CsA can inhibit OVA fibrillation in concentration dependent manner as revealed by Congo Red binding assay. The potential of CsA to inhibit amyloid formation was further corroborated by Congo Red binding assay^40^. In general, shaking of OVA solution for different time intervals at 25 °C resulted in marked increase in Congo Red binding as specified by increase in absorbance in comparison to Congo Red alone. Fig. 5 shows OVA fibrillation kinetics as a function of OVA fibril bound Congo Red. The increase in absorbance can be correlated with synthesis of OVA amyloid fibrils with exposed hydrophobic patches (Fig. 5A). Further, red shift in absorbance maxima of Congo Red (from 490 nm to 540 nm) also corroborates with generation of amyloid fibril structure with abundant cross β-sheet. The co-incubation of OVA with CsA (100 nM) during fibril synthesis, resulted in inhibition of absorbance peak intensity of OVA-amyloid bound dye (Fig. 5B). The decrease in absorbance intensity was more pronounced in presence of 500 nM of CsA (Fig. 5C). Besides decrease in absorbance, the treatment with CsA was also successful in nullifying the Congo Red mediated red shift in OVA fibril-Congo Red complex in dose dependent manner.

### CsA induces conformational changes in model protein OVA

**Figure 6:**
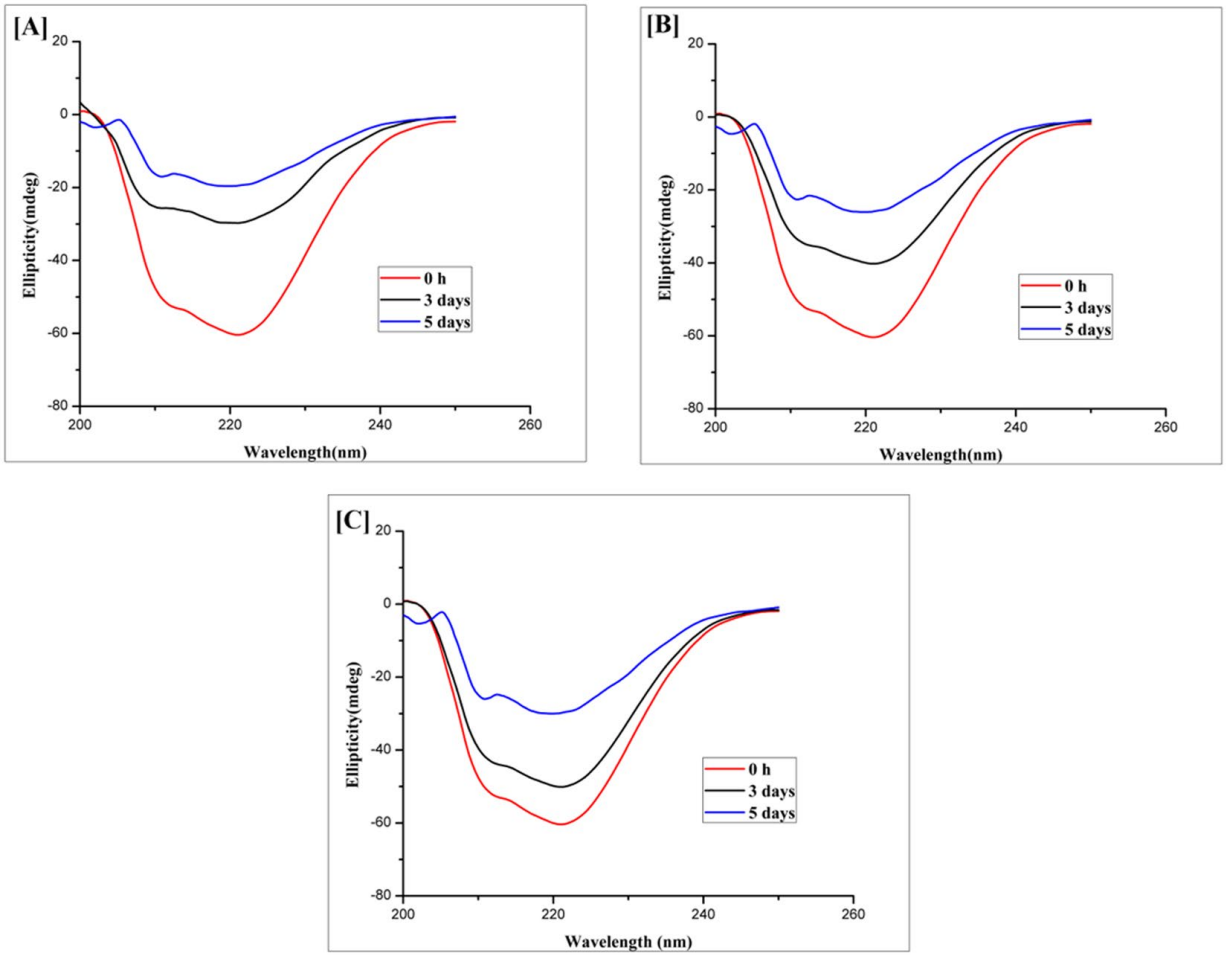
Effect of CsA on OVA amyloid synthesis as elucidated by by Far-UV CD spectroscopy. (**A**) Synthesis of OVA amyloid (15 µm) generated at 120 h time point. OVA aggregate formation in presence of 100 nM (**B**) or 500 nM (**C**) of CsA.

In order to detect the change in the secondary structure of OVA upon fibril formation, far UV region (200–250) CD spectroscopy was carried out^41^. CD spectra of OVA at 0 h post incubation with CsA correspond to native structure of the protein. As shown in Fig. 6A, post 120 h of co-incubation with CsA at 25 °C resulted in a negative peak at around 218 nm suggesting alpha helix to beta sheet structural transition in the OVA protein. Keeping into consideration the fact that fibril formation leads to change in MRE value at 218 nm, we assessed the effect of CsA on fibril synthesis employing far UV CD polarimeter studies. The presence of CsA (Fig. 6B,C) retarded structural conversion from alpha helix to beta sheet as evident by decrease negative band at around 218 nm as compared to native OVA. The observed CD data indicate that CsA inhibits OVA aggregation by decreasing the beta sheet content in as-formed fibril.

### CsA incurs change in morphology of as-formed OVA aggregate as revealed by TEM

**Figure 7:**
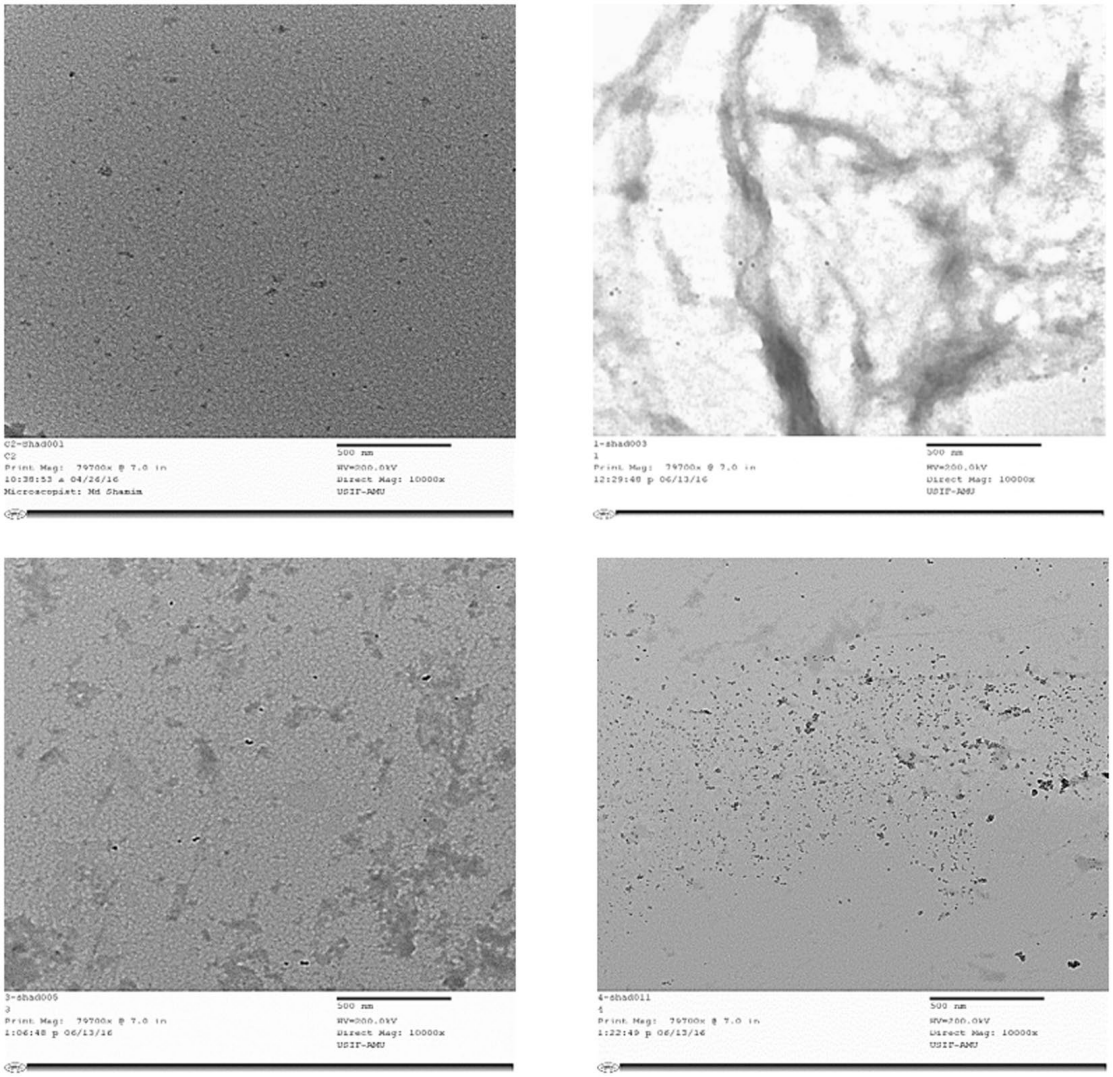
Effect of presence of CsA on synthesis of OVA fibril as elucidated by TEM analysis. (**A**) OVA solution incubated at room temperature (without stirring) served as control. (**B**) As- synthesized OVA amyloid generated after 5 days incubation. (**C**) Partial inhibition of OVA aggregation when OVA solution was stirred (induction of amyloid synthesis) in presence of 100 nM CsA for 5 days. (**D**) Almost complete inhibition of fibril synthesis when OVA fibril synthesis was executed in presence of 500 nM CsA for 5 days.

TEM was used to study the effect of CsA on morphology of mature as-formed OVA fibril. It is evident from electron micrograph that OVA-amyloid sample possessed long branched entities which were supposed to be characteristic feature of a protein fibril. The presence of CsA during synthesis of OVA-fibril resulted in short and sparsely populated non fibrillar aggregates (Fig. 7). The TEM analysis further suggests that incubation of OVA with CsA can inhibit OVA aggregation and resulted in significant alteration in the morphology of OVA aggregates as well.

### CsA alleviates OVA-fibril mediated cytotoxicity in neuroblastoma cells

**Figure 8:**
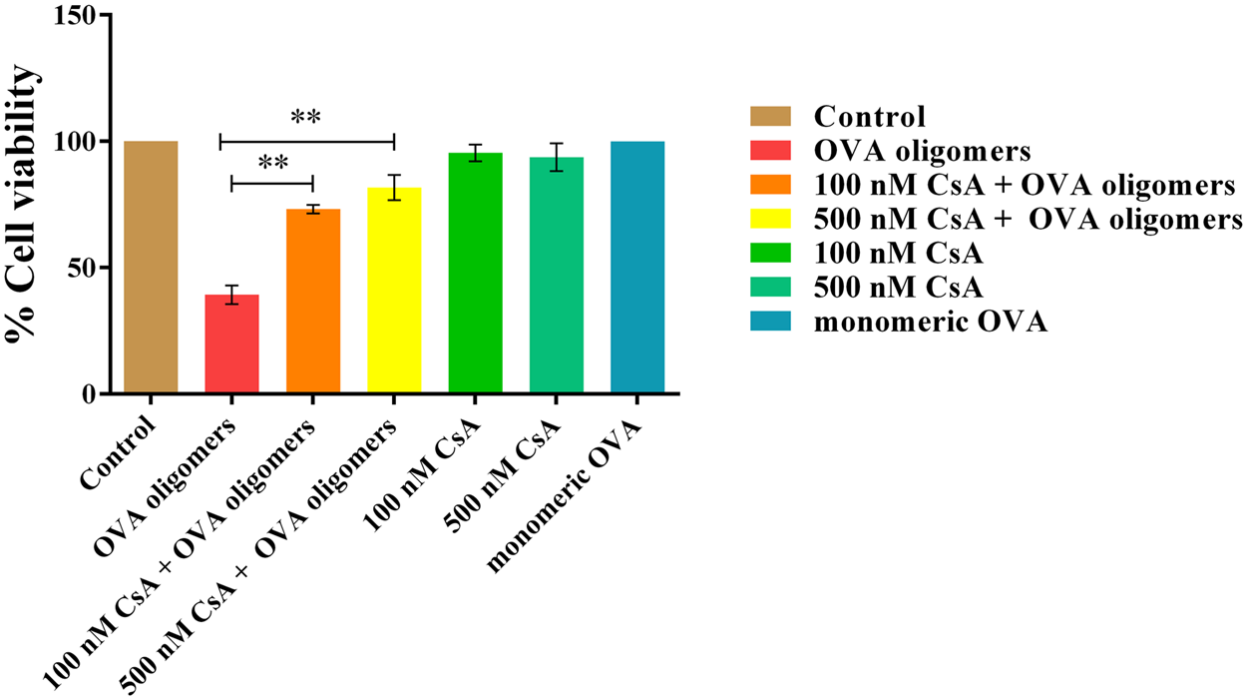
Cyclic peptide CsA alleviates OVA amyloid mediated cytotoxicity against neuroblastoma SH-SY5Y cells. Cell cytotoxicity of exponential phase intermediates of OVA amyloid fibril, synthesized in the presence of CsA (500 nM) on SH-SY5Y cell lines. The exposure to neat CsA (100 and 500 nM) did not incur any cytotoxicity to the cells.

SH-SY5Y (human neuroblastoma cell line) cells were exposed to intermediates species of OVA (at a corresponding monomer concentration of 1 mg/ml) fibrillation in presence of increasing concentration of CsA. The extent of OVA-fibril cytotoxicity was ascertained by employing MTT assay^42^ (Fig. 8). The OVA intermediate species caused approximately 40% death of the treated cells. The co-incubation of lag phase OVA fibril with CsA reverted amyloid induced toxicity to the cells. Cell toxicity of OVA may be attributed to the amyloid mediated disruption of the cell membrane. Further, incubation of SH-SY5Y cells pre-exposed to OVA intermediated with CsA failed to revert back incurred toxic manifestations. This observation rules out involvement of any other protective mechanism apart from aggregation inhibition (data not shown) in CsA mediated alleviation of cytotoxicity in neuroblastoma cells. Cell viability was rescued to 73% and 82% in presence of 100 nM and 500 nM CsA respectively. This observation establishes that increase in cell viability was due to anti amyloidogenic behavior of CsA.

## Discussion

Protein fibrillation has been loosely considered to be a multi-step process involving several or at least more than one quasi-static equilibria between monomers and subcritical or critical nuclei. This is generally followed by an essentially irreversible conversion of critical nuclei to mature fibrils^43,44^. Within this framework, the kinetics of fibrillation can be manipulated at both first as well as second quasi-equilibria in a range of manners. Recently various innovative tools have been employed to dissect the chemical kinetics of protein aggregation process^39^. The highly reproducible ThT fluorescence based kinetic data of the present study can be exploited to define the OVA aggregation mechanism and also to decipher underlying molecular events^39^. We observed that once a minimal but critical concentration of OVA aggregates had been generated through primary nucleation of monomers, surface-catalyzed secondary nucleation became the dominant process. We speculate that the surface of the newly formed dimers/trimers and other quasi species served as catalytic site that eventually help in the generation of toxic oligomeric species^45^. The as–synthesized oligomers can grow and transform into mature fibrils, thus promoting the formation of additional toxic species^46^.

Critical analysis of chemical kinetics suggests that various potential chemical entities generated during fibril formation could inhibit both primary nucleation as well as other secondary pathways^47^. Besides monomers/oligomers, these molecules seem to have potential to bind to both surface as well as terminal ends of growing fibril^48^. For example, removal of monomer could result in a decrease in the rate of various events (i.e. primary as well as secondary nucleation, and elongation), at molecular level^49^. It has also been observed that removal of oligomers affects both primary as well as secondary nucleation. On the other hand, binding of inhibitor causes reduction in both elongation as well as secondary nucleation process depending on whether these molecules bind to the surface or to the terminal ends of the protofibril.

The inhibition of specific microscopic steps involved in OVA aggregation is expected to have subjective effects on the generation of toxic oligomers^50^. More specifically, inhibition of primary nucleation should profoundly delay aggregation reaction without affecting the total load of toxic oligomers generated during the fibrillation. In fact, an alteration in the number of oligomeric species can be correlated with the suppression of either elongation or secondary nucleation steps of the process. The above specified two processes represent interchangeable pathways, as inhibiting elongation is expected to redirect the aggregation reaction toward secondary nucleation, that consequently increase the number of toxic oligomers, and vice versa. It seems either mature OVA monomers (that have been primed to enter into next stage intermediates), or the as-formed oligomers, both have tendency to bind to CsA. In fact, the cyclic peptide CsA has potential to bind intermediates and other elusive structures formed during lag phase. This leads to a situation where any of the above specified CsA bound OVA population remain unavailable for its transformation to mature amyloid. As depicted in Fig. 2A, CsA inhibits fibrillation process in the model protein OVA when added to reaction mixture in the beginning of the lag phase. The observed effect followed an analogous pattern to that of epigallocatechin-3-gallate (EGCG) which caused fibril inhibition in Chicken Cystatin^51^.

**Figure 9:**
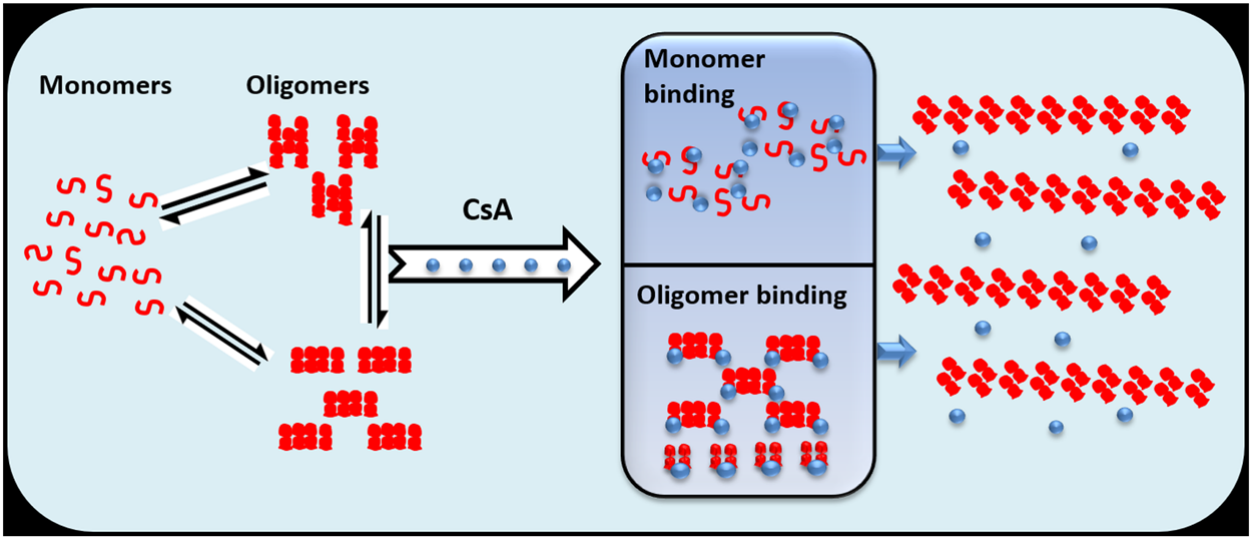
Schematic illustration depicting inhibition of OVA fibrillation by CsA. The model depicts CsA mediated inhibition of OVA fibrillation. More specifically, it highlights that cyclic peptide CsA affects nucleation step of OVA fibrillation mainly, while the elongation step remains largely unaffected.
II. **Binding of CsA with oligomeric intermediates as well as protofibril**: The cyclic peptide, CsA, may interact with OVA oligomers formed en route, and also binds to the surface of formed protofibril, thereby leading to depletion of sub and near-critical oligomer population. It may also inhibit binding of incoming monomers or oligomers on the surface of protofibril. CsA binding ensues in reduction of oligomer concentration in the solution that may in turn affect the nucleation step. Moreover, the oligomeric population that binds to CsA is unlikely to participate in fibril formation. It seems CsA binding can also imparts steric hindrance thus inhibit elongation process by blocking sites for attachment of incoming monomer on the preformed aggregates.

On the basis of results obtained in the present study, we have proposed, two naturally operative models to understand the effect of CsA on fibrillation process:

I. **Specific binding of CsA with monomeric precursor protein:** In general, critical nuclei are generated during fibrillation process. The formed nuclei have potential to eventually transform into mature fibril (Fig. 1A). One possible mechanism operative in CsA mediated fibril inhibition could be its specific binding to monomeric form of the protein. CsA trapped monomers disturb the monomer-oligomer equilibrium, which seems to be re-compensated by dissociation of early formed aggregates (Fig. 9). Binding of CsA with OVA oligomers coerce fibril formation. Beside inhibiting the primary nucleation, the drug CsA has potential to modulate secondary nucleation pathway as well.

It should be noted that either of the above discussed operative modes can reduce number of oligomers and modulate synthesis of fibril as well.

Inhibition of amyloid synthesis upon co-incubation of CsA with parent protein suggests that there is a strong and specific interaction of CsA with both monomeric as well as oligomeric forms of the protein. This eventually causes inhibition of fibril growth. It can also be speculated that CsA inhibits amyloid formation by blocking newly generated binding sites in growing fibril. This in turn will not alow addition of incoming monomers. This would lead to depletion of the sub and near critical oligomers and eventually to partial blockade of the kinetic pathway until the CsA surface gets saturated or generation of new nuclei occurs. Irrespective of what comprehensive kinetic mechanism involved, the distinct inhibitory role of CsA reported in the present study has remained conspicuous. Interestingly, CsA was found to inhibit fibril formation of Aβ amyloid as well (Fig. S1). The Aβ amyloid fibrillogenesis has direct correlation with various neurodegenerative complications including Alzheimer’s disease.

In next set of the study, we demonstrated that CsA successfully alleviates amyloid induced neuronal toxicity in SH-SY5Y cells. This could be otherwise considered as the ability of CsA to diminish amyloid generation. The cell toxicity is generally caused by soluble non β sheet oligomers formed during fibrillogenesis^52^. The data of the present study demonstrates that CsA extends or increases lag phase of amyloid formation. This in turn suggests that CsA has potential to target early aggregate species of the amyloid synthesis cascade. The observed effect could be correlated to CsA mediated sequestering of toxic oligomers so that such intermediates are no more available to contribute synthesis of amyloids. On the other hand, CsA by itself showed no cytotoxic effect when allowed to interact with SH-SY5Y cells. The observed results suggest that the non fibrillar aggregates formed in the presence of CsA do not harm the neuronal cells. It can be speculated that CsA and other related compounds may have therapeutic relevance against amyloids related diseases^53–56^.

On the basis of the data of the present study, we can infer that CsA with close end cyclized amino acid residues and specific surface chemistry, is capable of modulating both the onset as well as progression of OVA fibrillation process *in vitro*. The involved inhibition can be correlated with binding of CsA with monomer and intermediates states of the model protein OVA. The crucial steps involve monomer depletion (and subsequent shifting of the fibril synthesis equilibrium toward precursor) and/or trapping of sub- and near-critical nuclei (and subsequent kinetic blockade until the surface is saturated). The observation may have great deal of implications and form the basis of future studies to elucidate the effect of CsA mediated inhibition of fibrillation process at molecular level. It can be argued that in the present experimental set up the model protein OVA was allowed to interact with CsA without competition from other proteins which is quite unlikely to any realistic situation prevailing in natural system. Nevertheless, the study opens interesting avenues to setup future strategies and to have better insight regarding mechanism involved in deciphering role of cyclic peptides in inhibition of amyloid formulation.

Our preliminary data on CsA mediated inhibition of Amyloid β fibril formation correlate potential of cyclic peptide CsA in alleviation of Alzheimer’s disease. The observation may have great impact in regulating fibril induced cytotoxicity in other related diseases as well. Finally, we conclude that CsA mediated amyloid inhibition may have great deal of implications in discovery of agents that interfere with the fibrillation based health issues in human beings.

## Materials and Methods

### Chemicals and reagents

The Cyclosporin A, Thioflavin T, Congo Red and Ovalbumin used in the present experiments were purchased from Sigma-Aldrich (St.Louis, MO) unless otherwise specified. Aβ-42 was kind gift from Dr. Imtaiyaz Hasan of JMI, New Delhi, India. Anti-OVA monoclonal antibody 3G2E1D9 was purchased from Santa Cruz Biotechnology, Inc.3-[4,5-dimethylthiazol-2-yl]-2,5-diphenyl tetrazolium bromide (MTT) was obtained from Amresco,USA. Human neuroblastoma cell line (SH-SY5Y) was procured from American Type Culture Collection (ATCC, USA).

### Preparation of OVA aggregates

OVA was dissolved in 25 mM Tris buffer, 50 mM NaCl, and 1 mM DTT, pH 7, carrying 0.01% azide aggregation buffer at a concentration of 1 mg/ml and incubated at room temperature under continuous agitation (at 90 rpm). The synthesis of OVA fibril was established by Thioflavin T (ThT) and Congo Red (CR) binding assays. The formed fibrils were scanned to study their physical attributes using Transmission Electron Microscope (TEM).

### Thioflavin T (ThT) fluorescence kinetics

Thioflavin T (ThT) binding assay was carried out to quantify OVA amyloid formation and also to examine the impact of CsA on this process. ThT fluorescence assay has been widely used to detect cross -β sheet structure of amyloids. The growth of amyloid formation is accompanied by building of a cross-β sheet structure, that readily binds with ThT and eventually ensues in a significant rise in fluorescence intensity.

Briefly, OVA aggregates (100 μg) obtained at various time points were incubated with 30 μM ThT solution (30 μl of 1 mM ThT stock solution) at room temperature. ThT specific amyloid fluorescence was measured on Hitachi F-4500 fluorescence spectrophotometer. The sample was excited at 450 nm and spectra were recorded at wavelength range of 450 nm to 600 nm. The excitation and emission slit widths were fixed at 5 nm and 10 nm respectively.

### Effect of seeding on OVA fibrillation Kinetics

OVA solution (1 mg/ml) was incubated at room temperature with fibrillation buffer under continuous agitation (90 rpm). An aliquot of the preformed aggregated seed was added (stated as percentage of monomer concentration) to the incubation mixture in presence of CsA as per specification of experimental setup. The aggregation of OVA in presence of preformed seeds was monitored ex situ. The control setup was carried out as parallel experiment that was also spiked with preformed fibril seed in absence of CsA.

#### Fluorescence microscopic study

The interaction of ThT with OVA aggregates was also followed employing fluorescence microscopy as described elsewhere^57^. OVA aggregates prepared in presence of varying amount of CsA allowed to interact with ThT and mounted on coverslip. The formed fibrils were imaged by fluorescence microscope (Zeiss AxioVision, Germany).

### Congo Red (CR) binding study

A stock solution (1 mM) of CR in ethanol was prepared. The working CR solution (20 μM) was prepared in PB (pH 7.4) using CR stock solution (1 mM). The CR solution was mixed with 100 μg of aggregates and incubated for 30 min at 25 °C. UV absorbance was measured in the spectral range 300–700 nm using Perkin Elmer UV/VIS spectrometer model lambda 25.

### Circular dichroism (CD) spectroscopy

Circular dichroism study was performed using quartz cell with 0.1 cm path length in JASCO spectropolarimeter (J-815) instrument. The temperature was maintained at 25° ± 1 °C using Peltier Thermostat with Multitech water circulator. The instrument was calibrated using D-10-camphorsulfonic acid. The scan speed of 100 nm/min and response time of 2 s was used for spectra collection. Each sample was scanned in the range of 200–250 nm with final protein concentrations being 200 μg/ml.

### Transmission electron microscopy (TEM)

TEM studies were performed using JEOL transmission electron microscope operating at an accelerating voltage of 200 kV. The amyloid formation was probed using 200-mesh copper grid covered by the carbon-stabilized formvar film. Excess of fluid was removed after 2 min and the grid was then negatively stained with 2% (w/v) uranyl acetate. Images were viewed at 10,000X.

### Western blot analysis of OVA oligomers inhibition

Exponential phase of OVA fibrillation was fractioned in 10% SDS-polyacrylamide gels, transferred to polyvinylidene difluoride membranes, and incubated with 3G2E1D9 monoclonal antibody. After incubation and stipulated washing steps, the membrane was incubated with horseradish peroxidase conjugated goat-anti-mouse antibody (1:5000) for 1 hr at 37 °C. Finally, post washing, the bands onto the membrane were developed by enhanced chemiluminescence (ECL) using ECL kit, BioRad.

### MTT Cell Viability Assay

The MTT assay was employed to determine the viability of SH-SY5Y neuroblastoma cells^41^. Briefly, SH-SY5Ycell line was allowed to attain 70% confluency in DMEM in 96-well plates. The cells were washed with HBSS followed by treatment with early OVA oligomers (at a corresponding monomer concentration of 1 mg/ml) synthesized in presence of CsA for 24 h at 37 °C under 5% CO_2_ respectively_._ The cells were washed to remove excess of OVA oligomers and further incubated in fresh media for 24 h. For viability assay, the cells were washed twice and treated with 20 µl of MTT solution (5 mg/mL in PBS). The cells were incubated for additional 4 h at 37 °C, and the medium was carefully aspirated. Finally, the cells were solubilized in 200 µL of DMSO, and the absorbance of purple formazan was measured in each well at 570 nm. The control cells without prior exposure to the fibril solutions were used as positive control.

### Statistical analysis

All data were presented as mean ± standard deviation from 3 independent determinations. The statistical analysis was made by performing one-way ANOVA for 3 independent determinations. The significance of results was determined as p ≤ 0.01, unless otherwise stated.

## Electronic supplementary material

Supplementary Information
