# Supplementary material for: Cyclic undecapeptide Cyclosporin A mediated inhibition of amyloid synthesis: Implications in alleviation of amyloid induced neurotoxicity
Source: Sci Rep. 2018 Nov 23;8:17283. doi: 10.1038/s41598-018-35645-4 (PMC6251898; doi:10.1038/s41598-018-35645-4)
Supplement: Supplementary file 1 — Supplementary Information [file 41598_2018_35645_MOESM1_ESM.pdf]

Title: Cyclic undecapeptide Cyclosporin A mediated inhibition of amyloid synthesis: Implications in alleviation of amyloid induced neurotoxicity

Authors: Shadab Kazmi[1], Anzar Abdul Mujeeb[1], Mohammad Owais[1]

Affiliations: [1]Molecular Immunology Laboratory, Interdisciplinary Biotechnology Unit, Aligarh Muslim University, Aligarh-202002, INDIA.

Figure-S1 Effect of presence of CsA on kinetics of Aβ-42 amyloidogenesis. ThT fluorescence spectra of Aβ-42 was determined to see the effect of presence of CsA after incubation at 37 °C The fibril synthesis of Aβ-42 was allowed to proceed in presence of increasing concentration of CsA (100 and 500 nM). Absorption fluorescence spectra of ThT bound mature Aβ-42 amyloid species generated. The effect of presence of CsA on generation of Aβ-42 amyloid was monitored by plotting absorption fluorescence spectrum of mature OVA amyloid formed at 8 h time point. The native ova failed to bind with ThT dye. The CsA inhibited Aβ-42 amyloid synthesis in dose dependent manner.

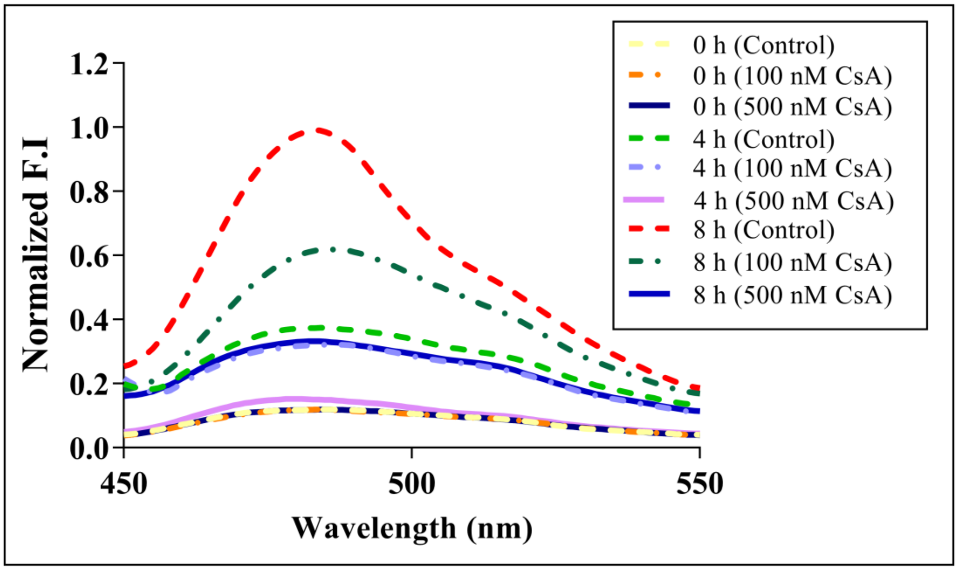

Figure-S1

Figure-S2 **Western blot profile of amyloid intermediates.** The western blot profile of various amyloid intermediates. Western blot analysis of OVA-amyloid probed with OVA specific 3G2E1D9 monoclonal antibodies reveals presence of monomeric, dimeric as well as oligomeric OVA (lane i) formed during start of growth phase of OVA aggregation (without CsA). Lane ii shows inhibitory effect CsA (500 nM) on the formation of monomeric and dimeric forms of OVA . Unmarked lane showed 200μm and 100μm OVA intermediates without CsA.

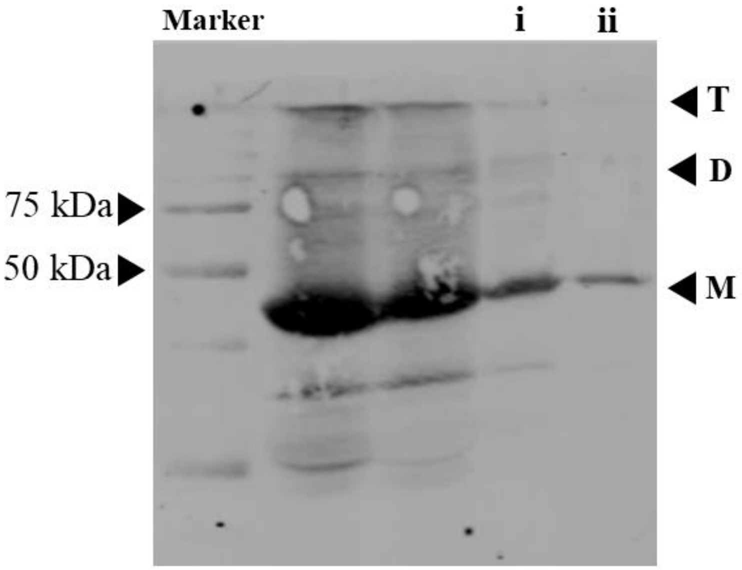

Figure-S2
